# Gastroesophageal Reflux Disease (GERD) and Risk of Incident Acute Myocardial Infarction: A Systematic Review and Meta‐Analysis of Cohort Studies

**DOI:** 10.1002/jgh3.70295

**Published:** 2025-10-13

**Authors:** Tinsae Anebo, Thitiphan Srikulmontri, Karecia Byfield, Elvis Obomanu, Phuuwadith Wattanachayakul, Michael Davis

**Affiliations:** ^1^ Department of Internal Medicine Jefferson Einstein Hospital Philadelphia Pennsylvania USA; ^2^ Department of Gastroenterology Jefferson Einstein Hospital Philadelphia Pennsylvania USA

**Keywords:** acute myocardial infraction (AMI), cardiovascular diseases, gastroesophageal reflux disease (GERD), risk factor

## Abstract

**Aims:**

Recent evidence suggests that gastroesophageal reflux disease (GERD) is associated with cardiovascular diseases, including increased coronary artery calcification. However, whether GERD is a risk factor for acute myocardial infarction (AMI) remains uncertain. This study aims to clarify this association through a systematic review and meta‐analysis.

**Methods and Results:**

We searched MEDLINE and EMBASE databases from inception to January 2025, including cohort studies that compared AMI incidence in individuals with and without GERD. Relative risk (RR), hazard ratio (HR), and 95% confidence intervals (CIs) were extracted and combined using a random‐effects model with the generic inverse variance method. Our meta‐analysis included six cohort studies with 1 324 362 participants. Patients with GERD had a 27% increased risk of incident AMI compared to those without GERD (pooled RR: 1.27, 95% CI: 1.13–1.47; *I* ^2^ = 88%, *p* = 0.0001). The funnel plot showed no significant publication bias. Potential mechanisms underlying this association include chronic inflammation, oxidative stress, and autonomic dysfunction.

**Conclusion:**

These findings suggest that GERD may contribute to AMI risk, highlighting the need for further research into preventive strategies and targeted interventions, such as acid suppression therapy and lifestyle modifications, to mitigate cardiovascular risk in GERD patients. Future studies should also explore mechanistic pathways to better understand this link and improve patient care.

## 1 Introduction

Gastroesophageal reflux disease (GERD) is a condition characterized by the backflow of stomach contents, causing bothersome symptoms and potential complications. While heartburn and regurgitation are the most common symptoms, GERD can also manifest in various ways, including chronic cough, globus sensation, wheezing, posterior laryngitis, dental erosion, and even idiopathic pulmonary fibrosis [1].

Globally, GERD affects approximately 13.3% of the population, with a higher prevalence of 15.4% in North America. In the United States, GERD imposes a substantial economic burden, with annual costs estimated at $10 billion. Established risk factors for GERD include being over 50 years old, smoking, NSAID use, obesity (BMI > 30), low socioeconomic status, and female sex. A US study found that 44.1% of participants had a history of GERD symptoms, 30.9% experienced symptoms in the past week, and 54.1% continued to have persistent symptoms despite daily proton pump inhibitor (PPI) use [1].

Acute myocardial infarction (AMI), commonly known as a heart attack, is diagnosed based on a rise and/or fall in cardiac biomarkers, particularly cardiac troponin levels, with at least one measurement exceeding the 99th percentile upper reference limit. This must be accompanied by evidence of myocardial ischemia, which may include ischemic symptoms, new or presumed new significant ST‐segment–T wave changes or new left bundle branch block, pathological Q waves on an electrocardiogram (ECG), imaging findings of new loss of viable myocardium or regional wall motion abnormalities, or the detection of an intracoronary thrombus via angiography or autopsy [2].

GERD and acute myocardial infarction have many overlapping interactions. GERD‐induced chest pain closely resembles cardiac pain, often leading to diagnostic confusion. Studies indicate that esophageal stimulation may trigger cardiac pain by inducing dysrhythmias or coronary spasm, thereby compromising coronary blood flow [3, 4, 5]. Studies have also shown that myocardial ischemia can worsen GERD by causing esophageal dysmotility or relaxation of the lower esophageal sphincter [4, 6, 7]. Recent evidence suggests that gastroesophageal reflux disease (GERD) is associated with cardiovascular diseases, including increased coronary artery calcification [8]. However, there is still conflicting evidence on whether GERD is a risk factor for developing acute myocardial infarction (AMI). Therefore, we aim to explore this association using all available data on GERD and the risk of incident AMI. Thus, we conducted a systematic review and meta‐analysis of observational studies examining the impact of GERD on the risk of developing Acute Myocardial Infarction.

## 2 Method

### 2.1 Search Strategy

Four investigators (T.A., P.W., K.B., E.O.) independently conducted searches in Medline and Embase databases from inception through January 2025. We searched for studies evaluating the association between gastroesophageal reflux disease (GERD) and myocardial infarction (MI) or acute coronary syndrome (ACS). For GERD, the following terms were used: the Medical Subject Heading (MeSH) “Gastroesophageal Reflux” and the text words “gastroesophageal reflux disease,” “gastro‐oesophageal reflux disease,” “GERD,” “acid reflux,” and “heartburn.” For myocardial infarction and ACS, we used the MeSH terms “Myocardial Infarction”, “Acute Coronary Syndrome”, and “Angina Pectoris”, together with the text words “myocardial infarction,” “MI,” “heart attack,” “acute coronary syndrome,” “ACS,” “ST‐elevation myocardial infarction,” “STEMI,” “non‐ST‐elevation myocardial infarction,” “NSTEMI,” and “angina.” These terms were combined using Boolean operators, with all GERD‐related terms grouped together and all myocardial infarction/ACS‐related terms grouped together, and both groups were then combined using “AND” to identify studies that addressed both domains. No language restrictions were applied. The same investigators independently assessed the eligibility of the retrieved records, with further discussions involving another investigator (T.S.) to resolve any conflicts. Abstracts and unpublished studies were excluded.

### 2.2 Eligibility Criteria

The eligibility criteria were as follows: Included studies must be observational studies (i.e., case–control or cohort) published as original research, assessing the association between GERD and risks of incident acute myocardial infarction. Studies must include a group of individuals with GERD and another group without GERD and must provide effect estimates representing the association between GERD and the risk of incident acute myocardial infarction in the form of odds ratios (OR), relative risk (RR), or hazard ratios (HR), accompanied by 95% confidence intervals (CIs) or survival curves. Raw data adequate for calculation for the effect sizes is acceptable. As the eligibility criteria did not uniformly specify adjustments for confounders (e.g., smoking, obesity, diabetes), which could undermine validity, we addressed this by extracting adjusted effect estimates whenever possible and prioritizing the use of adjusted risk ratios in the meta‐analysis. This approach was taken to reduce residual confounding and enhance the robustness of our findings.

### 2.3 Data Extraction

We employed a standardized data collection protocol to extract the following information: last name of the first author, study country, study design, publication year, total number of participants, recruitment protocol, GERD diagnosis, AMI diagnosis, follow‐up duration, baseline characteristics and comorbidities, and variables adjusted for in multivariate analysis. Data extraction was conducted independently by four reviewers (T.A., P.W., K.B., E.O.), with a fifth author (T.S.) serving as an adjudicator to resolve discrepancies through discussion and consensus. Two investigators (T.A. and P.W.) also applied the Newcastle‐Ottawa Scale for cohort studies to evaluate research quality, focusing on the quality of participant recruitment, comparability between groups, and accuracy of outcome ascertainment [9]. To further ensure robustness, we prespecified subgroup analyses based on key methodological factors (e.g., adjustment for confounders) and clinical characteristics, provided that a sufficient number of studies were available, and we planned sensitivity analyses to explore sources of heterogeneity.

### 2.4 Statistical Analysis

Data analysis was conducted using Review Manager 5.3 software from the Cochrane Collaboration. Point estimates with standard errors from each study were combined using DerSimonian and Laird's generic inverse variance method [10]. Due to heterogeneous background populations and protocols among the studies, a random‐effects model was employed. Statistical heterogeneity was assessed using Cochran's Q test, supplemented by *I* ^2^ statistics to quantify the proportion of total variation across studies attributable to heterogeneity rather than chance. *I* ^2^ values categorize heterogeneity as insignificant (0%–25%), low (26%–50%), moderate (51%–75%), or high (> 75%) [10]. To assess publication bias, funnel plots were generated when sufficient studies were available, and if more than 10 studies were included, Egger's regression test was additionally performed [9].

## 3 Results

**FIGURE 1:**
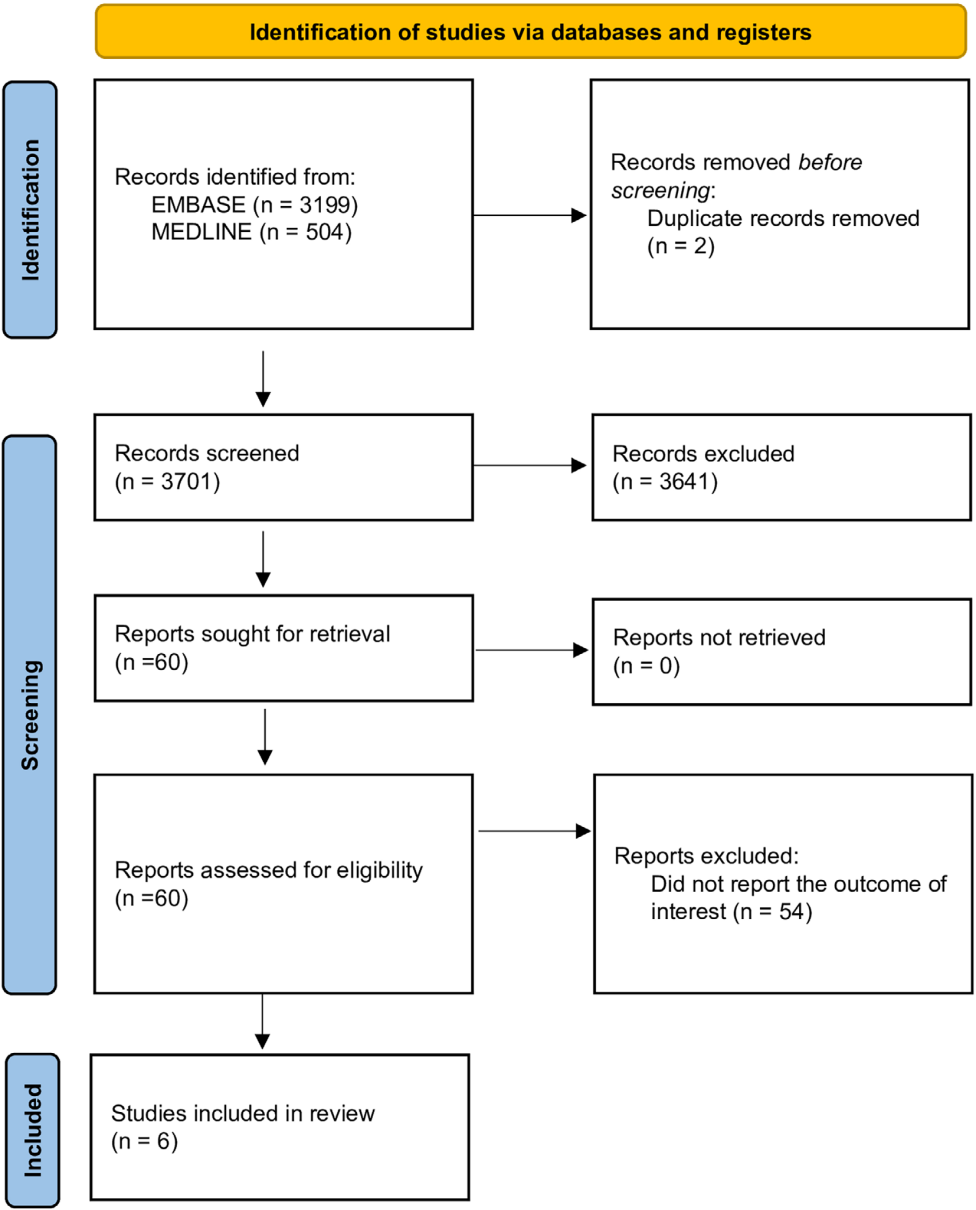
PRISMA flow diagram. Flowchart summarizing study identification, screening, eligibility assessment, and inclusion for the meta‐analysis of gastroesophageal reflux disease (GERD) and acute myocardial infarction (AMI). A total of 3703 records were identified (3199 from EMBASE and 504 from MEDLINE). After the removal of two duplicates and the exclusion of 3641 irrelevant studies by title and abstract, 60 full‐text articles were assessed for eligibility. Fifty‐four were excluded for lacking relevant outcome data, leaving six cohort studies for quantitative synthesis.

Our search strategy identified 3703 studies, with 3199 from EMBASE and 504 from MEDLINE. After removing two duplicates, we reviewed 3701 studies by title and abstract, excluding 3641 that did not meet the eligibility criteria related to study design, participants, or article type. Subsequently, we thoroughly reviewed 60 articles and excluded 54 for not reporting the relevant outcome. Ultimately, six studies met the eligibility criteria for our meta‐analysis [10, 11]. Figure 1 illustrates our search methodology and selection process, and Table 1 details the characteristics and quality assessment of the included studies.

**TABLE 1:** Main characteristics of the cohort studies included in the meta‐analysis.

	Chen et al.	Sun et al.	Eisa et al.	Lei et al.	Jansson et al.	Johansson et al.
Year of publication	2023	2022	2020	2017	2008	2003
Country of origin	United Kingdom	United Kingdom	United States	Taiwan	Norway	United Kingdom
Study design	Prospective cohort study	Mendelian randomization study	Retrospective cohort study	Retrospective cohort study	Case–control study	Retrospective cohort study
Total number of participants	All: 330 751 GERD: 20 583 Non‐GERD: 310 168	All: 367 441 GERD: 78 707 Non‐GERD: 288 734	All: 587 200 GERD: 200 400 Non‐GERD: 386 800	All: 326 284 GERD: 54 422 Non‐GERD:271 862	All: 65 333 GERD: 3153 Non‐GERD: 40 210	All: 17 084 GERD: 7084 Non‐GERD: 10 000
Recruitment of participants	Participants were recruited from the UK Biobank study, which included over 500 000 individuals aged 40–69 years from 22 centers across the UK between 2006 and 2010, without baseline cardiovascular disease. Individuals with and without gastrointestinal symptoms (GIs) were followed until the onset of incident cardiovascular disease.	Participants were recruited from the UK Biobank study and the QSKIN study.	Participants were recruited from the Explorys database, which includes data from the US population.	Participants were recruited from the Taiwan National Health Insurance Research Database between January 1, 2000, and December 31, 2011.	Participants were recruited from a Norwegian county's health survey database from 1995 to 1997. The survey included 70% of eligible county residents, with data collected through self‐reported questionnaires and physical examinations.	Participants were recruited from the General Practice Research Database in the UK. All patients with a first recorded diagnosis of GERD in 1996 were identified.
Diagnosis of GERD	GERD was identified using ICD‐9 and ICD‐10 codes from the nationwide inpatient dataset, primary care dataset, and cancer registries. The accuracy of these ICD codes has been validated in the annual report of the Audit Commission for Local Authorities of the National Health Service in England and Wales.	GERD was identified in the UK Biobank study using a combination of self‐reported GERD symptoms, ICD‐9 and ICD‐10 diagnoses, and GERD‐related medication use.	GERD was identified using CPT codes for patients who had an EGD within 1 week of diagnosis.	GERD was identified using ICD‐9 codes 530.11 or 530.81. The Bureau of National Health Insurance mandates that GERD patients must be diagnosed via endoscopy or 24‐h pH monitoring before PPIs can be prescribed.	GERD was identified using a questionnaire about heartburn or acid regurgitation symptoms in the past 12 months. Participants who reported severe symptoms of recurrent heartburn or acid regurgitation were classified as having GERD.	GERD was identified manually through the database, including the first ever code for GERD, acid reflux, acid regurgitation, esophagitis, and heartburn. A questionnaire was then sent to the corresponding general practitioner to confirm the GERD diagnosis based on clinical history and tests performed.
Diagnosis of AMI	AMI was defined using ICD‐9 and ICD‐10 codes from the nationwide inpatient dataset, primary care dataset, and cancer registries.	AMI was identified using Phenocode from the FinnGen consortium (released on May 11, 2021), which contains integrated genetic data and disease trajectories.	AMI was identified using CPT codes related to CAD‐AMI. Cases with CAD or AMI prior to the GERD diagnosis were excluded to ensure the primary outcome occurred after the GERD diagnosis.	AMI was identified using the ICD‐9‐CM codes 410.XX, 410.0–410.6, 410.7, or 410.9.	AMI was identified through a self‐reported questionnaire asking: “Do you have/have you had a myocardial infarction?”	AMI was identified manually using a computerized profile with a diagnosis code of myocardial infarction during follow‐up.
Follow‐up duration	Median: 11.8 years	N/A	N/A	11 years	N/A	4 years
Average age of participants (years)	All: 55.1 ± 8.1	N/A	GERD: 67 Non‐GERD: 77	GERD: 51.6 ± 16.9 Non‐GERD: 51.4 ± 16.9	N/A	N/A
Percentage of male	All: 42.2%	N/A	GERD:52.1% Non‐GERD: 51.8%	GERD: 46.5% Non‐GERD: 46.4%	All: 31.1% GERD: 49% Non‐GERD: 47%	N/A
Variables adjusted in multivariate analysis	Age, gender, Townsend deprivation index, body mass index, education, race, smoking status, drinking status, physical activity, adherence to a healthy diet, diabetes, hypertension, and hyperlipidemia.	N/A	Age, gender, hypertension, hyperlipidemia, smoking, diabetes, obesity, PPI therapy	Age, gender, hypertension, diabetes, hyperlipidemia, congestive heart failure, ischemic stroke, PPI therapy	Age, sex, tobacco use, BMI, and socioeconomic status	Age, sex, calendar year, smoking status, BMI, alcohol consumption, previous chest pain, ischemic heart disease, heart failure, hypertension, diabetes, hyperlipidemia
Newcastle‐Ottawa score	Selection: 4 Comparability: 2 Outcome: 2	Selection: 4 Comparability: 1 Outcome: 1	Selection: 4 Comparability: 1 Outcome: 3	Selection: 4 Comparability: 1 Outcome: 2	Selection: 4 Comparability: 1 Outcome: 2	Selection: 4 Comparability: 1 Outcome: 2

Abbreviations: AMI, acute myocardial infarction; BMI, body mass index; CAD, coronary artery disease; CPT, current procedural terminology; GERD, gastroesophageal reflux disease; ICD‐10, International Classification of Diseases, 10th Revision; ICD‐9, International Classification of Diseases, 9th Revision; ICD‐9‐CM, International Classification of Diseases, 9th Revision, Clinical Modification; NHANES, National Health and Nutrition Examination Survey; PPI, proton pump inhibitor; UK Biobank, United Kingdom Biobank.

**FIGURE 2:**
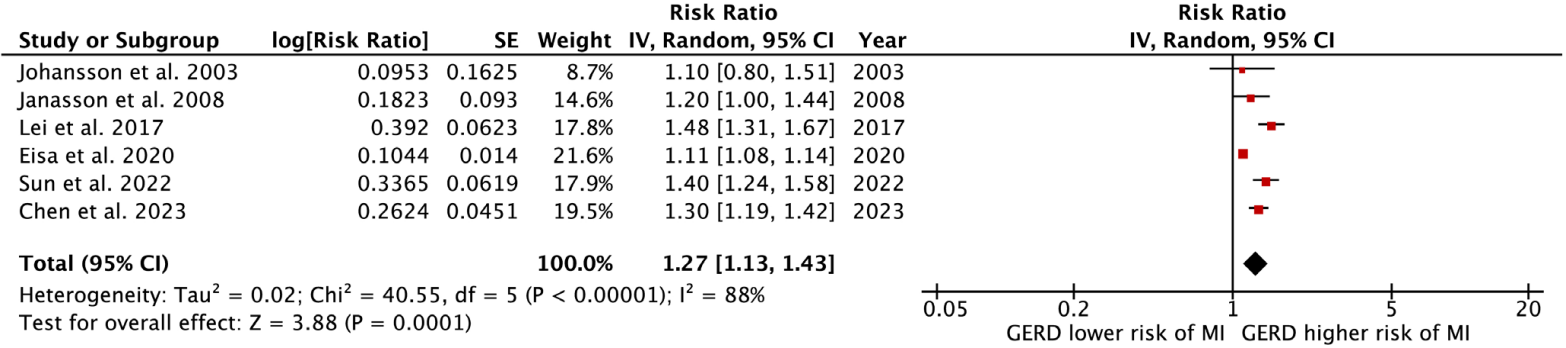
Forest plot of the association between GERD and incident AMI. Forest plot showing the pooled relative risk (RR) of incident AMI in individuals with GERD versus controls. Overall pooled RR = 1.27 (95% CI, 1.13–1.43; *p* = 0.0001), indicating a statistically significant increased risk of AMI among GERD patients. Random‐effects model used. High heterogeneity observed (*I* ^2^ = 88%).

**FIGURE 3:**
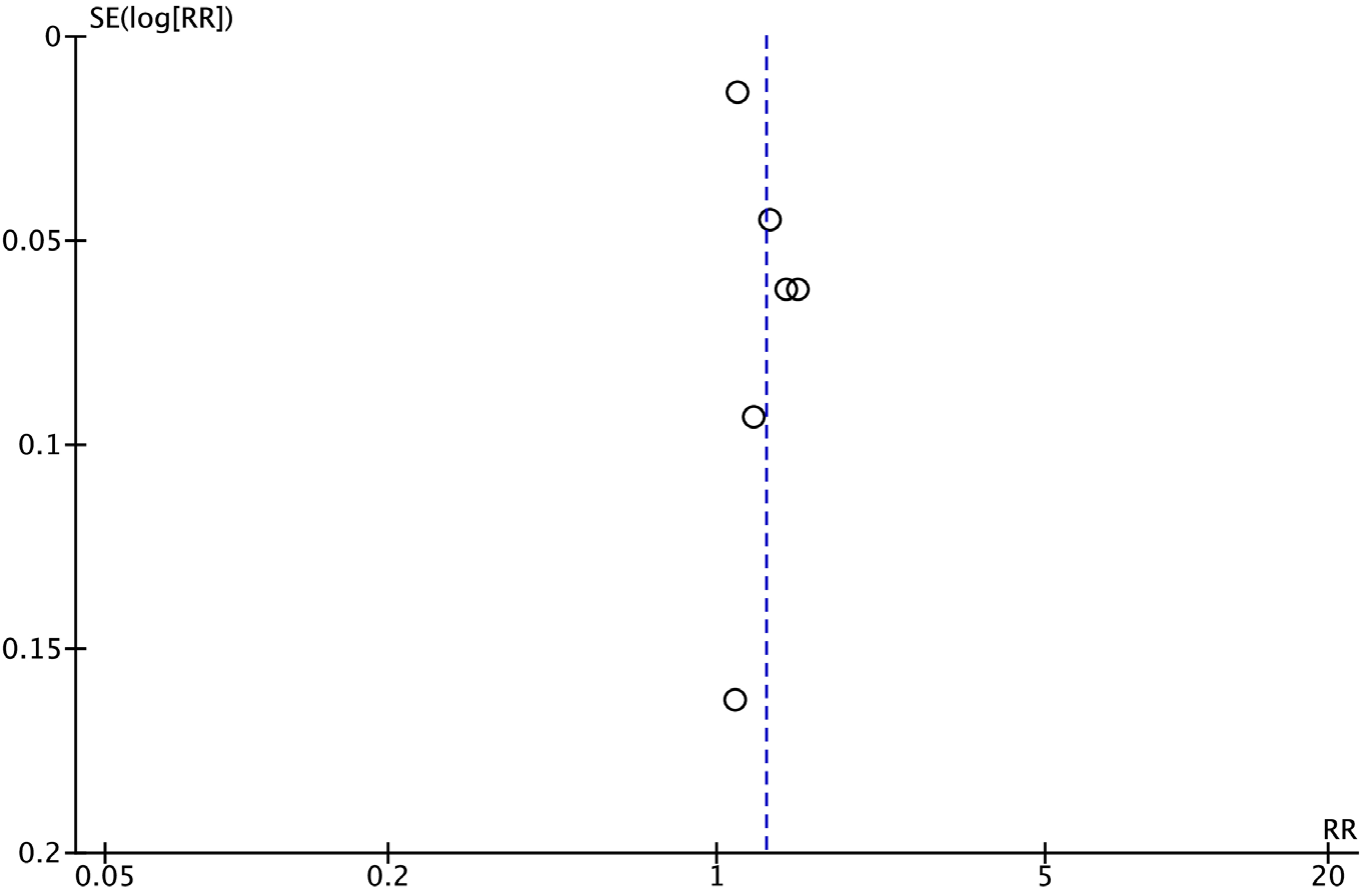
Funnel plot for publication bias assessment. Funnel plot evaluating publication bias among the six included studies. The symmetrical distribution of studies around the pooled effect estimate suggests no evidence of significant publication bias.

Six cohort studies involving 1 324 362 participants examined the association between GERD and the incidence of AMI. Pooled analysis demonstrated a higher risk of incident AMI among individuals with GERD compared to those without (pooled RR: 1.27, 95% CI: 1.13–1.43, *p* = 0.0001; Figure 2). Notably, high statistical heterogeneity was observed (*I* ^2^ = 88%). Given the inclusion of six studies in this meta‐analysis, publication bias was assessed, and the funnel plot showed no evidence of bias (Figure 3).

## 4 Discussion

To the best of our knowledge, this is the first systematic review and meta‐analysis evaluating the relationship between GERD and incident AMI. Our analysis revealed that patients with pre‐existing GERD had a modestly increased risk of developing AMI (RR = 1.27, 95% CI: 1.13–1.43, *p* = 0.0001) compared to those without GERD.

Several mechanisms may underline this association. First, GERD and coronary artery disease (CAD) share multiple common risk factors. For example, Song et al. demonstrated that male gender, alcohol use, smoking, elevated body mass index, and metabolic syndrome increase the risk of both conditions, potentially elevating MI risk [12]. Second, diagnostic misclassification may occur; some patients diagnosed with GERD might have underlying CAD. GERD is often diagnosed based on clinical evaluation and therapeutic trials prior to invasive investigation, yet more than half of patients with ischemic heart disease lack a history suggestive of CAD, and gastrointestinal history has limited predictive value [13]. Third, inflammation associated with GERD may contribute to coronary atherosclerosis. Both erosive and non‐erosive forms of GERD have been linked to elevated inflammatory markers, including IL‐1β, IL‐6, IL‐8, IL‐10, IFN‐γ, TNF‐α, MMP‐3, and MMP‐9—which play key roles in CAD pathogenesis [14]. For instance, IL‐6 promotes inflammatory cell infiltration into atheromatous plaques, while MMPs and TNF‐α facilitate collagen degradation, increasing plaque vulnerability [15]. Fourth, a neural reflex may reduce coronary perfusion in response to esophageal acid exposure. Although direct pathophysiological evidence is limited, clinical studies have shown that esophageal acidification can decrease coronary blood flow in patients with epicardial or microvascular coronary disease and those with coronary vasospasm [16, 17]. Finally interestingly several studies have explored the impact of PPIs, commonly used to treat GERD, on cardiovascular risk. Shah et al. found that PPI use in GERD patients was associated with a 1.16‐fold increased risk of myocardial infarction (95% CI, 1.09–1.24). Additionally, Ghebremariam et al. identified that PPIs elevate plasma levels of asymmetric dimethylarginine (ADMA), an inhibitor of nitric oxide synthase, which can impair endothelial function and increase cardiovascular risk [18, 19].

These findings have several practical implications and suggest directions for future research. First, regarding diagnosis, a history of GERD may lead to anchoring bias when evaluating patients with chest pain, given the overlapping symptoms of GERD and acute MI. Considering the elevated MI risk in this population, clinicians should pursue comprehensive diagnostic evaluations and close monitoring, and future research might focus on developing a clinical risk score for these patients. Second, in terms of treatment, although the interaction between omeprazole and clopidogrel is well established, the impact of PPIs on MI risk and overall cardiovascular outcomes remains uncertain. While some evidence suggests that GERD treatment may alleviate ischemic electrocardiographic changes, the effects on major adverse cardiovascular events and mortality have not been thoroughly investigated [16]. Future studies should explore these outcomes across various treatment modalities, including pharmacologic, endoscopic, and surgical interventions.

This meta‐analysis has several limitations. Most included studies employed matched case–control designs using population‐based datasets, which provided large sample sizes for assessing the association between GERD and AMI. However, it remains unclear whether these studies adequately controlled for the comorbidities common to both conditions. Additionally, since most studies were conducted in Europe and Asia, further research is needed to determine whether these findings are generalizable to populations with different characteristics. Another limitation of our study is the exclusion of abstracts and unpublished data, which may introduce the potential for publication bias. Another limitation is the absence of standardized diagnostic criteria for GERD and MI across the included studies. Some relied on ICD‐9/10 codes, others on self‐reported symptoms, or a combination of both. This variability in case definition may reduce diagnostic accuracy and contribute to heterogeneity in the pooled estimates. Lastly, another limitation is the significant heterogeneity observed across studies (*I* ^2^ = 88%), which may reduce the reliability of the pooled estimates. This heterogeneity likely reflects differences in study design, geographic region, diagnostic definitions of GERD and MI, and the range of confounders adjusted for. Because fewer than 10 studies were available [9], we were unable to perform meta‐regression to formally investigate sources of heterogeneity.

## 5 Conclusion

Our study revealed that GERD is associated with a higher incidence of AMI. Further research is needed to understand the underlying mechanisms, evaluate preventive strategies, and develop targeted interventions to mitigate this risk and improve patient care.

## Ethics Statement

The authors have nothing to report.

## Consent

The authors have nothing to report.

## Conflicts of Interest

The authors declare no conflicts of interest.

## Data Availability

The data that support the findings of this study are available on request from the corresponding author. The data are not publicly available due to privacy or ethical restrictions.
